# Predictors of Symptom Increase in Subsyndromal PTSD Among Previously Deployed Military Personnel

**DOI:** 10.1093/milmed/usab034

**Published:** 2021-02-13

**Authors:** Robyn M Highfill-McRoy, Jordan A Levine, Gerald E Larson, Sonya B Norman, Emily A Schmied, Cynthia J Thomsen

**Affiliations:** Leidos, San Diego, CA 92106-3521, USA; Health and Behavioral Sciences Department, Naval Health Research Center, San Diego, CA 92106-3521, USA; Leidos, San Diego, CA 92106-3521, USA; Health and Behavioral Sciences Department, Naval Health Research Center, San Diego, CA 92106-3521, USA; Health and Behavioral Sciences Department, Naval Health Research Center, San Diego, CA 92106-3521, USA; National Center for PTSD, 215 North Main St., White River Junction, VT 05009, USA; VA Center of Excellence for Stress and Mental Health, San Diego, CA 92161-0002, USA; UCSD School of Medicine, La Jolla, CA 92093-0602, USA; SDSU School of Medicine, San Diego, CA 92115, USA; Health and Behavioral Sciences Department, Naval Health Research Center, San Diego, CA 92106-3521, USA

## Abstract

**Introduction:**

Subsyndromal PTSD (sub-PTSD) is associated with functional impairment and increased risk for full PTSD. This study examined factors associated with progression from sub-PTSD to full PTSD symptomatology among previously deployed military veterans.

**Materials and Methods:**

Data were drawn from a longitudinal survey of Navy and Marine Corps personnel leaving military service between 2007 and 2010 administered immediately before separation (baseline) and ~1 year later (follow-up). Survey measures assessed PTSD symptoms at both times; the baseline survey also assessed potential predictors of symptom change over time. Logistic regression models were used to identify predictors of progression from sub-PTSD to full PTSD status.

**Results:**

Compared to those with no or few PTSD symptoms at baseline, individuals with sub-PTSD were almost three times more likely to exhibit full PTSD symptomatology at follow-up. Risk factors for symptom increase among those with sub-PTSD included moderate or high levels of combat exposure and utilization of fewer positive coping behaviors. Use of prescribed psychotropic medication was protective against symptom increase.

**Conclusion:**

This study identified several predictors of symptom increase in military veterans with sub-PTSD. Interventions targeting modifiable risk factors for symptom escalation, including behavioral and pharmacological treatments, may reduce rates of new-onset PTSD in this population.

## INTRODUCTION

PTSD is a significant concern among combat-exposed military personnel,^1^ especially in the aftermath of the Global War on Terrorism. In addition to those with PTSD, a substantial number of combat veterans have elevated PTSD symptoms but do not meet all criteria required for clinical diagnosis.^2,3^ Those who meet some but not all diagnostic criteria are often described as having partial, subclinical, or subsyndromal PTSD (sub-PTSD).^2,4^ Individuals with sub-PTSD exhibit functional impairment, physical health problems, and psychological distress at higher rates than healthy cohorts,^4,5^ though not as severely as those meeting criteria for full PTSD.^4,6,7^ Sub-PTSD has also been shown to be associated with increased risk for various psychiatric disorders, including major depressive disorder, panic disorder, agoraphobia, and, most notably, full PTSD.^6,8–11^ In prospective studies of both civilians and military personnel, sub-PTSD following trauma exposure strongly predicted later development of full PTSD.^12,13^

Although there is strong evidence that sub-PTSD is often a precursor to full PTSD, the underlying prevalence of the condition and mechanisms of symptom escalation require further examination. This is particularly critical in military populations facing combat exposure, which is the most common cause of PTSD among American men.^14^ Previous research on correlates of sub-PTSD has been largely cross-sectional in nature and thus unable to track the development of the disorder over time.^1,6,7,15–17^ The objectives of this longitudinal study were to (1) identify the baseline prevalence of sub-PTSD symptomatology in separating military service members previously deployed in support of the Global War on Terrorism, (2) examine risk and protective factors for both sub-PTSD and full PTSD in this population, (3) determine whether individuals with sub-PTSD at baseline were at heightened risk for screening positive for full PTSD at follow-up, and (4) identify factors related to symptom escalation between baseline and follow-up.

## METHODS

### Participants

Navy and Marine Corps service members enrolled in mandatory Transition Assistance Programs at 13 military installations nationwide were invited to complete a voluntary survey to assess physical and mental health issues in separating military personnel. All participants were preparing for honorable separation from active duty service. Baseline mail- and web-based surveys, administered between September 2007 and April 2010, assessed current PTSD symptoms as well as several demographic, military, and psychosocial factors. Factors were obtained using self-report measures and validated scales that were examined for internal consistency using Cronbach’s alpha coefficients. Follow-up surveys completed 6-12 months after baseline were used to assess changes in PTSD symptomatology. This study was approved by institutional review boards at the Naval Health Research Center and RTI International. Additional information about study procedures and survey development has been published previously.^18^

### Survey Measures

PTSD symptoms were measured at both baseline and follow-up using the validated PTSD Checklist—Civilian Version (PCL-C), a 17-item self-report measure corresponding to PTSD diagnostic criteria in the Diagnostic and Statistical Manual of Mental Disorders, Fourth Edition (DSM-IV) (α = .95).^1,2,19,20^ Respondents rated the degree to which they were bothered by each problem in the past month using a 5-point scale from 1 (not at all) to 5 (extremely). Items were summed to create a symptom severity score. Individuals were classified as having full PTSD symptoms if they had a score ≥50 and met criteria for all three symptom clusters (Criterion B: intrusive recollection, Criterion C: avoidance/numbing and Criterion D: hyperarousal). Consistent with previous research, individuals not meeting criteria for full PTSD symptoms were classified as having sub-PTSD symptoms if they met the definition for Criterion B and either (1) met the definition for Criterion C or Criterion D, or (2) reported at least one symptom from both Criterion C and Criterion D.^4,21,22^ Remaining participants were classified as having no/few PTSD symptoms. Responses on the follow-up survey were used to determine whether respondents who did not have full PTSD at baseline had (1) advanced to meet the criteria for full PTSD, or (2) experienced a clinically meaningful progression in symptoms (as denoted by a score increase of 10 or more points), indicating a significant decline in mental welfare regardless of full PTSD diagnosis status.^19,23^

Covariates from the baseline survey included demographic factors (age, sex, race/ethnicity, and current marital status), military service-related variables, and psychosocial measures shown to be associated with PTSD status in previous research.^4,5,8,15,24,25^ Service-related variables included service branch, combat exposure, and reason for separation. Combat exposure during the most recent deployment was assessed using 17 items from four previously developed scales describing potentially traumatic combat experiences (e.g., “I saw dead bodies or human remains”; “I personally fired my weapon at the enemy”) (α = .88).^1,18,26–28^ Respondents rated the frequency with which they experienced each event on a scale ranging from 1 (never) to 5 (51+ times). Responses were summed and trichotomized based on distribution of scores into none/low, moderate, and high combat exposure categories. Reasons for military separation included retirement, expiration of term of service, disability/physical problem, and other reasons, such as pregnancy/parenthood.

Baseline psychosocial measures included postdeployment social support and stressors, receipt of mental health counseling, prescription drug use, use of coping behaviors, depressive symptoms, health perception, and pain level.^4,5,8,15,24,25^ Social support and stressors were measured using scales from the Deployment Risk and Resilience Inventory.^18,26^ The 15-item social support scale assessed the extent to which family, friends, coworkers, and employers provided the respondent with emotional sustenance since returning from deployment (α = .82). Participants also reported the number of postdeployment stressors (i.e., natural disasters, physical assaults, and legal, occupational, or financial difficulties) they experienced in the past year using a 16-item scale. For both instruments, responses were summed and trichotomized based on distribution of scores into low, moderate, and high levels.

Mental health counseling, prescription drug use, and positive coping behaviors were measured using items from the 2005 Department of Defense Survey of Health Related Behaviors Among Active-Duty Military Personnel.^29^ Respondents were asked to report whether they sought mental health services (e.g., a mental health professional at a military facility, civilian mental health professional, or self-help group) in the past 12 months (yes/no). Individuals who answered “yes” were classified as having sought mental health counseling. Prescription drug information was ascertained through an item that asked if individuals had been prescribed medication for depression, anxiety, or sleeping problems. Response options included “yes, in the past 30 days,” “yes, in the past 12 months,” and “no.” Responses were dichotomized as “yes” or “no.” Coping behaviors were assessed using five items asking how respondents cope when they “feel pressured, stressed, depressed, or anxious.” Both positive behaviors (e.g., “saying a prayer” and “playing sports”) and negative behaviors (e.g., “smoking a cigarette” and “thinking about hurting myself”) were ranked on a 4-point scale (“frequently,” “sometimes,” “rarely,” or “never”). Items were summed to determine the number of positive coping behaviors used and then trichotomized (0-1, 2-3, and 4-5).

The Center for Epidemiologic Studies Depression Scale was used to measure current depressive symptoms (α = .79).^30–32^ Twenty items assessed the presence of depressive symptomatology in the last week, including sadness, hopelessness, loss of pleasure, and fatigue. Response options ranged from 1 (rarely or none of the time) to 4 (most or all of the time). Item responses were summed and dichotomized, using a standard cutoff score of 16 or higher to classify depressive symptoms as being present.

Current health perception and levels of bodily pain were assessed using items from the Medical Outcomes Study 20-Item Short Form Health Survey.^33,34^ Health perception was assessed by four items (“I am somewhat ill,” “I have been feeling bad lately,” “My health is excellent,” and “I am as healthy as anybody I know”), all of which were rated on a 5-point scale ranging from 1 (definitely true) to (definitely false). The latter two items were reverse scored, and an overall mean score was determined based on all responses, with a higher score indicating worse perceived health. If a respondent left blank two or more of the four items, the response was excluded from analyses. A separate item asked respondents how much bodily pain they had experienced in the past 4 weeks, with response options ranging from 1 (none) to 6 (very severe).

### Statistical Analysis

Baseline levels of each covariate were compared across the three symptom groups (no/few PTSD symptoms, sub-PTSD, and full PTSD) using chi-square tests of association for categorical variables and one-way analyses of variance for continuous variables. Post hoc chi-square testing (for categorical variables) and *t*-test analyses (for continuous variables) were then used to identify specific group differences. For participants without full PTSD at baseline, scores were compared between baseline and follow-up to determine whether their PTSD symptoms had significantly increased, as indicated by either meeting criteria for full PTSD (PCL-C score ≥50) or by experiencing a clinically meaningful progression in symptoms (PCL-C score increase ≥10). Finally, two logistic regression analyses were conducted to identify predictors of a clinically meaningful increase in PTSD symptoms for participants who did not have full PTSD at baseline. The first model compared those with sub-PTSD and those with no/few PTSD symptoms at baseline; the second, a subanalysis, included only those with sub-PTSD at baseline. In both cases, all covariates of interest were first entered into a univariable logistic regression model to examine statistical significance when considered in isolation. All covariates with a *P*-value <.10 were then entered into a multivariable logistic regression model, where a backward elimination approach was utilized to produce a final adjusted model including only covariates with a *P*-value <.05. All analyses were performed using SPSS software version 18.0.

## RESULTS

### Descriptive Analyses

The baseline survey was completed by 5,091 sailors and Marines who had deployed during their military service. Twenty-five percent (1,272) completed the follow-up survey 6-12 months later. Of those who completed the follow-up survey, 31 participants (1.9%) were excluded because they had not yet left military service. Demographic differences between eligible participants who completed the follow-up survey (*n* = 1,241) and those who only completed the baseline survey or were ineligible for follow-up (*n* = 3,850) were assessed using *t*-tests and chi-square tests of association. Nonresponders were disproportionately male, single, younger, non-Hispanic White, and likely to screen positive for full PTSD at baseline (results not shown). Branch of service was not related to follow-up survey completion.

Table I provides comparisons of participant characteristics across the three-symptom groups at baseline. In terms of demographic factors, individuals in both the sub-PTSD and full PTSD groups were younger than those in the no/few symptom group by an average of 3 years; however, the three groups did not significantly differ with regard to sex, race/ethnicity, or marital status.

**TABLE I.** Baseline Characteristics of Service Members as a Function of Baseline PTSD Status

	*n*(%)^a^		
	No/Few PTSD symptoms	Sub-PTSD symptoms^b^	Full PTSD symptoms^c^
	760 (61.2)	279 (22.5)	202 (16.3)
Sociodemographic factors			
Age, years, and mean (SD)^*^	31.6 (9.5)	28.3 (7.4)^†^	28.1 (7.8)
Sex			
Female	92 (12.1)	35 (12.5)	21 (10.4)
Male	666 (87.9)	244 (87.5)	180 (89.6)
Race/ethnicity			
Non-Hispanic White	516 (68.3)	196 (70.8)	125 (62.8)
Other	240 (31.7)	81 (29.2)	74 (37.2)
Marital status			
Single	248 (32.8)	103 (37.2)	67 (33.2)
Divorced/separated	67 (8.8)	31 (11.2)	28 (13.8)
Married	442 (58.4)	143 (51.6)	107 (53.0)
Service-related variables			
Service branch			
Navy	159 (20.9)	75 (26.9)	55 (27.2)
Marine Corps	601 (79.1)	204 (73.1)	147 (72.8)
Combat exposure level^*^			
None/low	327 (45.3)	87 (33.3)^†^	53 (27.3)
Moderate	239 (33.1)	87 (33.3)	49 (25.3)
High	156 (21.6)	87 (33.3)^†^	92 (47.4)^†^
Reason for separation^*^			
Retirement	258 (34.2)	46 (16.8)^†^	33 (16.7)
Expiration of term of service	382 (50.7)	170 (62.0)^†^	116 (58.6)
Disability/physical problem	24 (3.2)	15 (5.5)	15 (7.5)
Other	90 (11.9)	43 (15.7)	34 (17.2)
Psychosocial variables			
Postdeployment social support^*^			
Low	145 (20.2)	93 (35.2)^†^	103 (54.2)^†^
Moderate	246 (34.3)	110 (41.7)	62 (32.6)
High	326 (45.5)	61 (23.1)^†^	25 (13.2)^†^
Postdeployment stressors^*^			
Low	430 (56.7)	92 (33.1)^†^	33 (16.5)^†^
Moderate	257 (33.9)	108 (38.8)	70 (35.0)
High	71 (9.4)	78 (28.1)^†^	97 (48.5)^†^
Mental health counseling^*^			
No	630 (86.8)	191 (73.5)^†^	85 (46.2)^†^
Yes	96 (13.2)	69 (26.5)^†^	99 (53.8)^†^
Prescription drug use^*^			
No	720 (95.5)	243 (87.7)^†^	150 (75.8)^†^
Yes	34 (4.5)	34 (12.3)^†^^†^	48 (24.2)^†^
Positive coping behaviors^*^			
0-1	71 (9.4)	40 (14.4)	38 (18.9)
2-3	288 (38.1)	118 (42.6)	109 (54.2)
4-5	396 (52.5)	119 (43.0) ^†^	54 (26.9)^†^
Depressive symptoms^*^			
Not present	625 (85.6)	120 (43.3)^†^	17 (8.5)^†^
Present	105 (14.4)	157 (56.7)^†^	183 (91.5)^†^
Health perception and mean (SD)^*^	3.8 (0.9)	3.5 (0.9)^†^	2.8 (0.9)^†^
Pain level and mean (SD)^*^	1.7 (1.2)	2.2 (1.1)^†^	2.8 (1.1)^†^

Abbreviation: sub-PTSD, subsyndromal PTSD.

^a^ Some covariate totals do not match column totals because of missing data on specific items.

^b^ For post hoc testing, the no/few PTSD symptoms group is the reference for comparisons with the sub-PTSD group.

^c^ For post hoc testing, the sub-PTSD group is the reference for comparisons with the full PTSD group.

^*^ *P* < .01 for omnibus testing.

† *P* < .01 for post hoc testing.

Numerous military and psychosocial covariates significantly differed across the three-symptom groups. In terms of service-related factors, sailors were more likely than Marines to be in the sub-PTSD or full PTSD group. Consistent with previous research,^4,6,8^ risk factors were generally lowest among respondents with no/few PTSD symptoms, followed by those with sub-PTSD, and highest in those with full PTSD symptoms. This gradient suggests a clinically meaningful distinction between sub-PTSD and the other classification groups. Regarding service-related variables, just over one-fifth of those with no/few symptoms reported high combat exposure during deployment compared with one-third of those with sub-PTSD and almost one-half of those with full symptoms. Furthermore, the prevalence of respondents who reported retirement as their reason for military separation was more than twice as high among those with no/few symptoms as among both those with full PTSD and sub-PTSD symptoms, indicating that pre-retirement attrition was higher among the two PTSD groups.

The gradient effect across symptom groups was also evident in the prevalence of psychosocial covariates. Respondents with no/few PTSD symptoms were nearly twice as likely to report high postdeployment social support as those with sub-PTSD symptoms, and more than three times as likely to do so as those with full PTSD symptoms. Similarly, the use of numerous (4-5) coping behaviors was almost twice as prevalent among respondents with no/few symptoms compared with those reporting full symptoms, while those reporting sub-PTSD symptoms fell in the middle. This gradient pattern was also apparent regarding mental health counseling, prescription drug use, health perception, and pain level. Lastly, the prevalence of respondents who reported depressive symptoms was six times higher among those with full PTSD symptoms as among those with no/few PTSD symptoms; those reporting sub-PTSD symptoms again fell in the middle. This is consistent with previous research indicating that depression is often comorbid with both full and sub-PTSD disorder classifications.^9,16,35^

### Longitudinal Analyses

The next set of analyses examined change in PTSD symptoms from baseline to follow-up among participants who did not have full PTSD at baseline. The average change in PCL-C score over time significantly differed between respondents with sub-PTSD symptoms at baseline (mean increase of 1.7 points; SD = 14.8) and those with no/few symptoms at baseline (mean decrease of 3.5 points; SD = 12.0; *P* < .01; see Table II). Individuals with sub-PTSD at baseline were almost three times more likely to develop full PTSD symptoms at follow-up compared with those who reported no or few symptoms at baseline (20.4% and 7.2%, respectively; *P* < .01). Furthermore, respondents in the former group were almost five times more likely to experience a PCL-C score increase of 10 or more, indicating a clinically significant progression of PTSD symptoms, compared with those in the latter group (31.9% and 6.7%, respectively; *P* < .01).

**TABLE II.** Changes in PTSD Symptoms Over Time Among Service Members Who Did Not Have Full PTSD at Baseline

	6- to 12-Month Follow-Up		
	Mean PCL-C score change	New-Onset PTSD	PCL-C score increase by ≥10
Baseline	Mean (SD)	*n* (%)	*n* (%)
No/few PTSD symptoms (*n* = 760)	−3.5 (12.0)	55 (7.2)	51 (6.7)
Sub-PTSD symptoms (*n* = 279)	1.7 (14.8)^*^	57 (20.4)^*^	89 (31.9)^*^

Abbreviations: PCL-C, PTSD Checklist—Civilian Version; sub-PTSD, subsyndromal PTSD.

* *P* < .01.

The first multivariable logistic regression model (Model 1) revealed that the odds of reporting a clinically significant increase in PCL-C score at follow-up were 6.4 times higher among those with sub-PTSD compared to those with no/few symptoms at baseline (Table III). Moderate combat exposure and use of fewer positive coping behaviors were also shown to significantly increase the odds of symptom escalation. Respondents who reported moderate levels of combat exposure at baseline had more than twice the odds of reporting a clinically significant increase in PCL-C score at follow-up compared with those reporting no or low combat exposure. Similarly, respondents who reported utilizing the fewest (0-1) positive coping behavior at baseline had more than twice the odds of experiencing a clinically significant increase in symptom scores compared with those who reported using the most (4-5) positive coping behaviors.

**TABLE III.** Model 1: Multivariable Logistic Regression Model Predicting a Clinically Significant Increase in PTSD Score Among Those without Full PTSD at Baseline^a^^,^^b^

Characteristic	OR	95% CI
PTSD symptoms at baseline		
No symptoms	1.0	
Sub-PTSD	6.4^*^	3.2-8.1
Combat exposure level		
None/low	1.0	
Moderate	2.1^*^	1.2-3.6
High	1.4	0.8-2.5
Positive coping behaviors		
0-1	2.1^*^	1.3-3.2
2-3	1.3	0.7-2.6
4-5	1.0	

Abbreviations: OR, odds ratio; PCL-C, PTSD Checklist—Civilian Version; sub-PTSD, subsyndromal PTSD.

^a^ Additional variables that were originally entered into the backward elimination model include age, reason for separation, postdeployment social support, and depressive symptomatology.

^b^ This number includes only respondents who provided answers for all covariates entered into the final model (*n* = 899).

* *P* < .05.

A subanalysis (Model 2) examined the influence of baseline predictors of symptom escalation in only those with sub-PTSD at baseline. Similar to results for Model 1, combat exposure and lack of positive coping behaviors increased the odds of reporting a clinically significant increase in symptom score at follow-up (Table IV). Additionally, non-White race and female sex were associated with increased likelihood of symptom escalation, whereas the use of prescribed psychotropic medication was shown to be protective against significant symptom escalation in those with sub-PTSD at baseline.

**TABLE IV.** Model 2: Subanalytic Logistic Regressions Model Predicting a Clinically Significant Increase in PTSD Scores Among Those With Sub-PTSD at Baseline^a^^,^^b^

Characteristic	OR	95% CI
Combat exposure level		
None/low	1.0	
Moderate	2.5^*^	1.1-5.7
High	2.7^*^	1.2-6.3
Positive coping behaviors		
0-1	2.2^*^	1.1-4.5
2-3	1.6	0.6-4.1
4-5	1.0	
Prescription drug use		
No	1.0	
Yes	0.3^*^	0.1-0.9
Sex		
Male	1.0	
Female	2.7^*^	1.0-6.0
Race/ethnicity		
Non-Hispanic White	1.0	
Other	1.9^*^	1.0-3.7

Abbreviations: OR, odds ratio; PCL-C, PTSD Checklist—Civilian Version; sub-PTSD, subsyndromal PTSD.

^a^ Additional variables that were originally entered into the backward elimination model include postdeployment social support, stressors, and mental health counseling.

^b^ This number includes only respondents who provided answers for all covariates entered into the final model (*n* = 221).

* *P* < .05.

## DISCUSSION

The goals of this study were to document the prevalence, risk factors, and longitudinal course of sub-PTSD symptoms among military personnel transitioning to civilian life. Sub-PTSD was reported in 22.5% of baseline participants; 16.3% reported full symptoms and 61.2% reported no/few symptoms. Similar to previous studies,^4,6,8,36^ individuals with sub-PTSD symptoms at baseline differed from those reporting either full or no/few symptoms with respect to almost all military and psychosocial characteristics measured, which suggests a clinically meaningful distinction between sub-PTSD and both of the other symptom groups.

Different symptom courses were also observed for those with sub-PTSD versus no/few symptoms at baseline. The average symptom severity score for those with sub-PTSD symptoms increased by 1.7 points over the course of the study period, while the average score for those with no/few symptoms decreased by 3.5 points. Accordingly, respondents in the sub-PTSD group were almost three times more likely than those in the no/few symptom group to report full PTSD symptoms at follow-up. Differences between these two groups in psychosocial characteristics and symptom courses over time distinguish sub-PTSD as a clinically significant psychological condition in the absence of a formal diagnosis^4,6–8,36^ and may help practitioners recognize PTSD symptoms before they escalate further.

Multivariable modeling for the subsample of individuals with sub-PTSD at baseline identified combat exposure, female sex, and non-White race/ethnicity as risk factors for progression from sub-PTSD to full PTSD. On a more promising note, the use of positive coping behaviors and prescribed psychotropic medications was shown to be protective against PTSD symptom escalation. Secondary prevention efforts that focus on these two modifiable factors could be used as part of a formal treatment regimen after sub-PTSD symptoms have manifested to prevent the progression to full PTSD diagnosis. Furthermore, although coping behavior is considered a teachable skill set^37^ that is often a part of psychotherapy, it can also be delivered in nonclinical settings, such as through a class or seminar for veterans who are not engaged in a formal mental health program.

Several limitations and strengths of this study should be noted. The sample was comprised only of Navy and Marine Corps service members and cannot be generalized to other service branches. Furthermore, the sample only included service members who were honorably separating from the military and was therefore not representative of all military personnel. Potential systematic differences between those who did and did not complete the follow-up survey may have introduced a degree of response bias into the study. The patterns of non-response parallel those observed in prior military research; a 2015 RAND report concluded that technological, logistical, motivational, and trust-related factors may play a role in response patterns.^38^ However, a strength of the study is the inclusion of women and racial minorities, as had been suggested by previous research that did not include these groups.^3,4,7,17^

The study utilized the DSM-IV and corresponding PCL-C checklist for clinical diagnosis. As diagnostic criteria have since been modified with the publication of the DSM-V and updated PCL-5 checklist, further research is needed to determine if patterns observed in this study persist under the expanded and more granular criteria. This includes three additional items on strong negative beliefs including guilt, shame, anger, and distrust.^39^ The study also relied on self-reported measures and screening instruments, which, despite being validated in other samples and used extensively in military research, cannot be equated with clinical diagnoses and may lead to recall bias. Furthermore, PTSD is known to co-occur with other behavioral health issues that were not assessed in this study, such as substance misuse or dependence.^40^ Future research should examine the role of substance use in PTSD symptom trajectories. Lastly, given the tendency for psychiatric disorder symptoms to fluctuate over time,^41^ it must be considered that the sub-PTSD participants may have previously met full PTSD criteria at some point before the baseline assessment.

In this study, over 20% of personnel with sub-PTSD before separation developed symptoms consistent with full PTSD within the following year. Understanding sub-PTSD symptomatology and identifying modifiable factors that decrease the risk for symptom escalation, such as positive coping behaviors and prescribed psychotropic medication, are important steps in PTSD prevention and intervention efforts. Together with other literature suggesting that sub-PTSD is a clinically meaningful construct associated with psychosocial and physical impairment,^4,7,9,10,16,42^ the findings of this study highlight the need to assess, monitor, and treat sub-PTSD in previously deployed veterans.

## CONCLUSION

This study identified several risk factors for symptom increase in military veterans with sub-PTSD. Interventions targeting modifiable risk factors for symptom escalation, including behavioral and pharmacological treatments, may reduce rates of new-onset PTSD in this population.
